# Exome-Sequencing Identifies Novel Genes Associated with Recurrent Pregnancy Loss in a Chinese Cohort

**DOI:** 10.3389/fgene.2021.746082

**Published:** 2021-12-02

**Authors:** Huifen Xiang, Chunyan Wang, Hong Pan, Qian Hu, Ruyi Wang, Zuying Xu, Tengyan Li, Yezhou Su, Xu Ma, Yunxia Cao, Binbin Wang

**Affiliations:** ^1^ Reproductive Medicine Center, Department of Obstetrics and Gynecology, The First Affiliated Hospital of Anhui Medical University, Hefei, China; ^2^ NHC Key Laboratory of Study on Abnormal Gametes and Reproductive Tract (Anhui Medical University), Hefei, China; ^3^ Graduate School of Peking Union Medical College & Chinese Academy of Medical Sciences, Beijing, China; ^4^ Center for Genetics, National Research Institute for Family Planning, Beijing, China

**Keywords:** recurrent pregnancy loss, whole-exome sequencing, coagulation, FOXA2, KHDC3L

## Abstract

Recurrent pregnancy loss (RPL) is a common reproductive problem affecting around 5% of couples worldwide. At present, about half of RPL cases remained unexplained. Previous studies have suggested an important role for genetic determinants in the etiology of RPL. Here, we performed whole-exome sequencing (WES) analysis on 100 unrelated Han Chinese women with a history of two or more spontaneous abortions. We identified 6736 rare deleterious nonsynonymous variants across all patients. To focus on possible candidate genes, we generated a list of 95 highly relevant genes that were functionally associated with miscarriage according to human and mouse model studies, and found 35 heterozygous variants of 28 RPL-associated genes in 32 patients. Four genes (*FOXA2, FGA*, *F13A1*, and *KHDC3L*) were identified as being strong candidates. The *FOXA2* nonsense variant was for the first time reported here in women with RPL. FOXA2 knockdown in HEK-293T cells significantly diminished the mRNA and protein expression levels of LIF, a pivotal factor for maternal receptivity and blastocyst implantation. The other genes, with 29 variants, were involved in angiogenesis, the immune response and inflammation, cell growth and proliferation, which are functionally important processes for implantation and pregnancy. Our study identified several potential causal genetic variants in women with RPL by WES, highlighting the important role of genes controlling coagulation, confirming the pathogenic role of *KHDC3L* and identifying *FOXA2* as a newly identified causal gene in women with RPL.

## Introduction

Recurrent pregnancy loss (RPL), defined as two or more clinical pregnancy losses before 20 weeks of gestation, is a serious and complicated reproductive outcome (Practice Committee of the American Society for Reproductive Medicine 2020). It is estimated that <5% of women of reproductive age have experienced two spontaneous miscarriages and about 1% suffer three or more (Practice Committee of the American Society for Reproductive 2012). Although previous studies have revealed some causes of RPL, including parental and embryonic chromosomal abnormalities, endocrine disorders, uterine malformations and immunological disorders, the causes remain unexplained or are poorly understood in approximately half of the cases (Practice Committee of the American Society for Reproductive 2012; Saravelos and Regan 2014; El Hachem et al., 2017). In recent decades, several studies have suggested that genetic factors might play an important role in RPL (Page and Silver 2016; Pereza et al., 2017; Arias-Sosa et al., 2018; Quintero-Ronderos and Laissue 2019).

Previous variant screening and gene-association studies adopting a candidate gene strategy tried to unveil the genetic etiology of RPL and have identified numbers of potential causative genes and risk loci (Quintero-Ronderos and Laissue 2019). Deleterious variants of THBD, FOXD1, C4BP1, C3, WNT6, and KHDC3L have been detected in cases of RPL (Mohlin et al., 2013; Zhang et al., 2015; Quintero-Ronderos et al., 2017b; Mohlin et al., 2018; Quintero-Ronderos et al., 2019; Zhang et al., 2019), while polymorphisms of the genes encoding factor V Leiden (*FVL*), factor II (*F2*) and methylenetetrahydrofolate reductase (*MTHFR*), and others were found to increase the risk of RPL (Sergi et al., 2015; Yang et al., 2016; Pereza et al., 2017). Genome-wide association studies (GWAS), involving an unbiased genome-wide approach for identifying genetic determinants, have also been applied to identify risk loci for RPL (Li et al., 2010; Kolte et al., 2011). However, searching for candidate genes might have limited value in identifying the causative factors, considering that RPL is a highly genetically heterogeneous condition, whereas the loci identified by GWAS can only explain a very small proportion of the risk.

Whole exome sequencing (WES), a high-throughput technology, has now been exploited to identify causative genes and variants involved with RPL (Robbins et al., 2019). Pan e*t al.* (Pan et al., 2019) reported a consanguineous Chinese family with three women experiencing unexplained RPL and identified a rare homozygous frameshift variant of *CAPS* in all of them using WES. Exome sequencing in unrelated samples or single cases have also identified candidate maternal-effect genes, including PIF1, CCDC68, PLK1, MMP10, FLT1, PADI6, and FKBP4 (Quintero-Ronderos et al., 2017a; Qian et al., 2018; Demetriou et al., 2019; Maddirevula et al., 2020). In addition, several genes associated with fetal lethality (DYNC2H1, ALOX15, FOXP3, and CHRNA1) were identified by exome sequencing in tissues from miscarriages (Shamseldin et al., 2013; Qiao et al., 2016; Reichert et al., 2016). These findings have helped understanding the genetic causes of RPL. However, the genetic roles of some maternal-effect genes in women with RPL need validation and replication in more samples, and any biological relevance to RPL needs further explanation. Furthermore, most of these studies were performed in European ethnic groups and few were in East Asian ethnic groups, especially Chinese populations.

Here, we performed WES on a Han Chinese cohort of 100 unrelated women with RPL. We particularly tried to identify deleterious variants in a subset of 95 genes that are known as candidates causing RPL in human studies or are related to the phenotype of pregnancy loss in mouse models. Overall, we found six rare variants in four strong candidate genes (*KHDC3L*, *FGA*, *F13A1*, and *FOXA2*) and an additional 29 variants in 24 candidate genes. We document several newly identified candidate genes and also replicated the genetic roles of some genes identified in previous studies, thus providing more biomarkers for RPL.

## Materials and Methods

### Study Subjects

One hundred unrelated Han Chinese women with a history of RPL were recruited from the Center for Reproductive Medicine at The First Affiliated Hospital of Anhui Medical University, Anhui, P. R. China. The mean age at diagnosis was 28.2 years (range 21–41). RPL was defined as a history of at least two spontaneous abortions prior to the 20^th^ week of gestation according to the criteria of the American Society for Reproductive Medicine (Practice Committee of the American Society for Reproductive 2012). Patients with abnormal karyotypes, autoimmune disorders, endocrine disorders, uterine malformations or malignancies were excluded. All patients donated peripheral blood samples for the extraction of genomic DNA. The study was approved by the Ethics Committee of the National Research Institute for Family Planning, Beijing, P. R. China. Written informed consent was obtained from all subjects.

### Whole-Exome Sequencing and Bioinformatics Analysis

The exomes of all participants were captured by Agilent SureSelect Human All Exon V6 Enrichment kits (Agilent, Santa Clara, CA, United States ) and then sequenced on a NovaSeq platform (Illumina, San Diego, CA, United States ) according to the manufacturer’s guide. All reads were mapped to the human reference genome (hg19) using Burrows–Wheeler Alignment version 0.7.9a (http://bio-bwa.sourceforge.net). Single nucleotide variants (SNVs) and indels were detected using the Genome Analysis Toolkit version 3.5 (https://gatk.broadinstitute.org/hc/en-us), and then annotated by ANNOVAR (https://annovar.openbioinformatics.org/en/latest/user-guide/download/).

All variants were filtered according to the following criteria: i) missense, nonsense, frameshift, non-frameshift or splicing site variants; ii) variant frequency <0.1% in total and East Asian populations of the gnomAD v2.1.1 database (http://gnomad.broadinstitute.org). iii) All the missense variants were predicted to be deleterious by Sorting Intolerant From Tolerant software (SIFT; http://sift-dna.org), PolyPhen-2 (http://genetics.bwh.harvard.edu/pph2/) and Mutation Taster (http://www.mutationtaster.org); and iv) non-frameshift variants that should reside in conserved sites among eutherian mammals (see Supplemental Figure S1). Furthermore, the remaining variants were classified according to the American College of Medical Genetics and Genomics/Association for Molecular Pathology (ACMG/AMP) guidelines as pathogenic, likely pathogenic, with uncertain significance.

Next, we divided the 100 patients into two groups according to the severity of their miscarriage phenotype: two and more than two. We counted the numbers of the mutations following the above criteria identified in each group.

### RPL-Associated Gene Set Analysis and Variant Validation

To narrow down the subset of candidate genes, we generated an RPL-associated gene set comprising genes that were related to this phenotype from previous human and animal studies. Human studies reporting candidate genes by WES or Sanger sequencing were searched in PubMed (https://pubmed.ncbi.nlm.nih.gov), prior to April 30 2020, using the following terms: (recurrent pregnancy loss OR recurrent spontaneous abortion OR recurrent miscarriage OR habitual abortion) AND (mutation OR variant OR exome sequencing). Genes showing miscarriage-associated phenotypes in gene knockout or mutated mouse models were searched in the Mouse Genome Informatics (MGI) databases (http://www.informatics.jax.org) under the following phenotype terms: abnormal embryo attachment; failure of embryo implantation; impaired embryo implantation; abnormal miscarriage rate; abnormal decidualization; abnormal postimplantation uterine environment; endometrial inflammation; abnormal uterine receptivity; uterine inflammation; abnormal uterine environment; and uterine hemorrhage. Fetal lethality genes were excluded, and only maternal-effect genes were retained. Finally, 95 genes were included in the RPL-associated gene set (Supplemental Table S1). The presence of potential causal variants of RPL-associated genes was validated by Sanger sequencing using the primers listed in Supplemental Table S2. Next, we also counted the numbers of variants identified in each group. The statistical significance of the relationship of the number of variants in the patients and the number of miscarriages was evaluated using Chi squared tests and a *p* value < 0.05 was considered statistically significant.

### Cell Culture

Human embryonic kidney (HEK)-293T cells were cultivated in Dulbecco’s modified Eagle’s medium supplemented with 10% fetal bovine serum, 100 mg/ ml penicillin and 100 mg/ ml streptomycin and maintained in 5% CO_2_ in humidified air at 37°C.

### RNA Interfering and Target siRNA Screening

The siRNA set for FOXA2, including three pairs of siRNA-FOXA2, a positive control, a negative control (NC), and fluorescein amidite FAM-labeled NC, was purchased from Genepharma (Shanghai, P. R. China). For transient transfection, cells were seeded into 6-well plates (1×10^6^ cells/well) and were transfected with Lipofectamine™ 2000 transfection reagent (Invitrogen, Carlsbad, CA, United States ) according to the manufacturer’s protocol. After being incubated for 24 and 48 h cells were washed and harvested to test the mRNA and protein expression levels of *FOXA2*, respectively. Cells were seeded in 6-well plates and transfected with siFOXA2-375 and NC siRNA in the subsequent assays. The most effective FOXA2-siRNA: siFOXA2-375 duplexes were: forward 5′–CCA​UGA​ACA​UGU​CGU​CGU​ATT–3′ and reverse 5′–UAC​GAC​GAC​AUG​UUC​AUG​GTT–3′.

### Reverse Transcription-Quantitative Polymerase Chain Reaction

After transfection for 24 h, cells were collected, and total RNA preparations were extracted using TRIzol reagent (Invitrogen). We used a NanoDrop2000 spectrophotometer (Thermo Fisher Scientific, Waltham, MA, United States ) for assessment of RNA concentration and quality. Extracted RNA was reverse transcribed into complementary DNA with the P5X All-In-One MasterMix Kit (Abmgood, Vancouver, BC, Canada) according to the manufacturer’s instructions. Levels of mRNAs of *FOXA2* were measured in samples using SYBR premix ex Taq Kit (Takara, Tokyo, Japan) in an ABI Step One Real-Time system (Applied Biosystems, Waltham, MA, United States ). The relative expression of mRNA to that for β-actin was calculated using the 2^−∆∆Ct^ method. The primers used for RT–qPCR are listed in Supplementary Table S4.

### Western Blotting

After transfection for 48 h, total protein samples from cells were cleaved in RIPA lysis buffer (Applied Biosystems) and the protein concentration was determined using a bicinchoninic acid assay (Thermo Fisher Scientific, Waltham, MA, United States ); then, an equal amount of protein from each sample was loaded, separated using 10% sodium dodecyl sulfate polyacrylamide gel electrophoresis (Epizyme, Shanghai, P. R. China) and transferred to polyvinylidene fluoride membranes (EMD Millipore Corp., Billerica, MD, United States ). The membranes were blocked with 5% skim milk for 2 h, then added to a diluted primary antibody and incubated overnight at 4°C. And then with the second antibody, the membranes were incubated for 1.5 h at room temperature. The signal was detected using electrochemiluminescence kits (Amersham Biosciences, Piscataway, NJ, United States ). GAPDH was used as a reference.

## Results

### Clinical Descriptions

One hundred unrelated Han Chinese women with a history of 2–6 miscarriages were included in our study. Among them, 60, 30, and 10 patients had experienced 2, 3 and ≥4 miscarriages, respectively. Analysis of the association between the number of miscarriage and increasing age group (28.05 ± 3.25 years and 28.53 ± 2.85 years, respectively) showed that there was no significantly difference in age between the two groups (*p* = 0.692). None of the patients have had a successful full-term pregnancy except for one who reported a livebirth. All patients had normal karyotypes.

### Whole-Exome Sequencing Analysis

**FIGURE 1:**
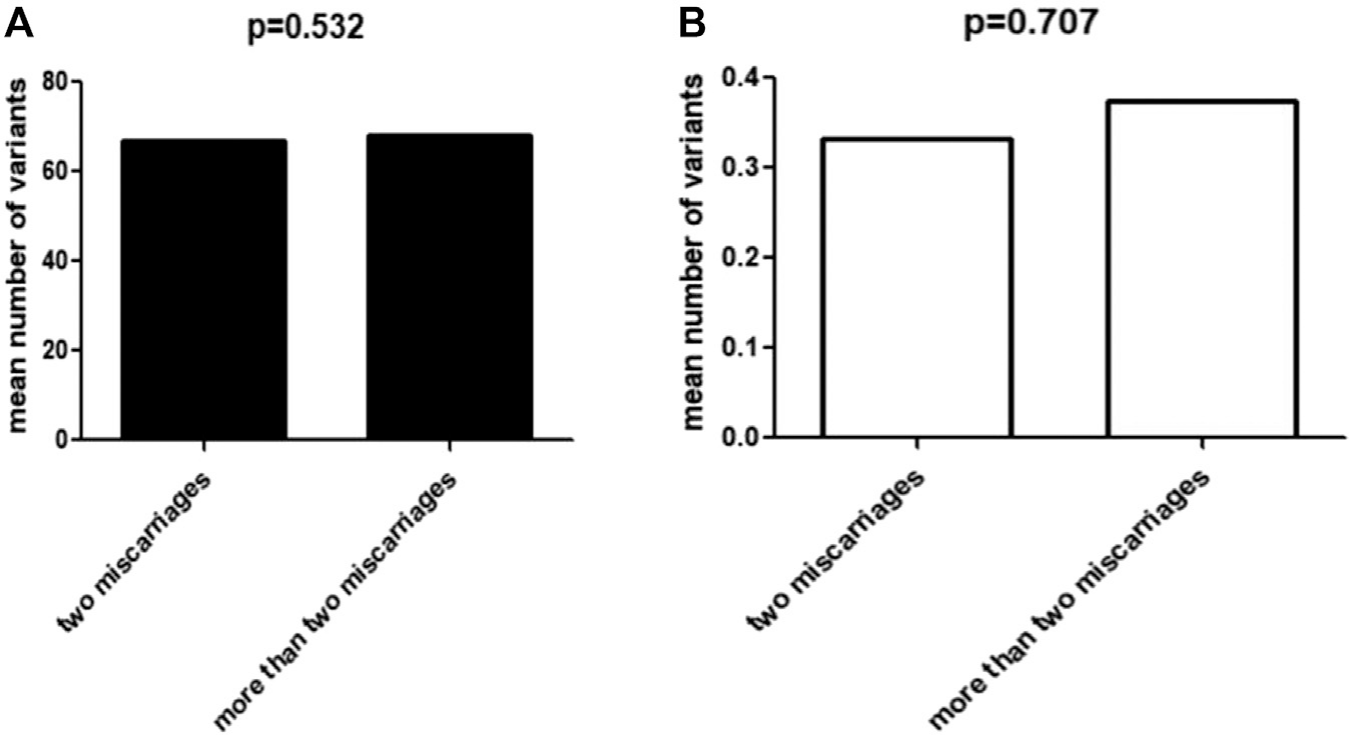
Correlation between miscarriage phenotype and variant accumulation. **(A)** The mean number of total deleterious variants in two groups with miscarriages and more than two miscarriages, respectively. **(B)** The mean number of deleterious variants of RPL-related genes in two groups with miscarriages and more than two miscarriages, respectively.

After frequency filtering (Supplemental Table S3), a total of 117,620 nonsynonymous variants fulfilling the frequency criteria from 100 exome-sequenced patients were obtained and a mean of 1176 ± 142 variants per sample were kept for the following *in silico* evaluation. Of these rare variants, 4929 missense variants were predicted deleterious by all online programs (SIFT, PolyPhen2, Mutation Taster), 1161 variants (frameshift, nonsense, splicing site) might cause loss of function, and 646 were non-frameshift insertion/deletions. Thus, each patient had on average 67.4 rare, deleterious nonsynonymous variants and 11.6 potential loss-of-function variants. Furthermore, we divided the 100 patients into two groups according to the severity of their miscarriage phenotype: two miscarriages and more than two miscarriages, including 60 and 40 patients, respectively (Figure 1A). Analysis of the distribution of these variants (4013 and 2723, respectively, Figure 1B) showed that there was no significantly different distribution between the two groups (*p* = 0.532).

### Identification of Potential Causal Variants in the RPL-Associated Gene Set

**FIGURE 2:**
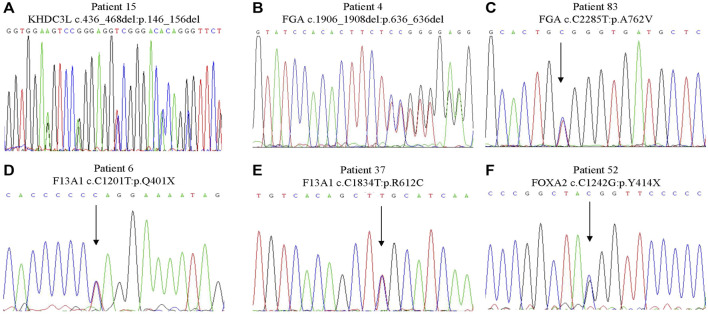
Sanger sequencing of the variants in strong candidate genes. **(A)** KHDC3L c.436_468del: p.146_156del. **(B)** FGA c.1906_1908del: p.636_636del. **(C)** FGA c.C2285T:p.A762V. **(D)** F13A1 c.C1201T:p.Q401X. **(E)** F13A1 c.C1834T:p.R612C. **(F)** FOXA2 c.C1242G:p.Y414X.

Among the large number of rare, deleterious nonsynonymous variants across exomes, we found 35 variants in 28 out of 95 RPL-associated genes, including 26 missense and three nonsense variants, one frameshift and five non-frameshift deletions. We prioritized six variants of four genes as potential causal variants in the light of previous human genetic studies and phenotypes in mouse models mimicking RPL (Table 1). The other 29 variants of 24 genes were considered as candidates for causing RPL because the knockout of these genes in mice resulted in miscarriage-associated phenotypes. Furthermore, analysis of the distribution of these 35 variants (20/60 and 15/40, respectively) showed that there was no significantly different distribution between the two groups (*p* = 0.707).

(i.) FOXA2 ***.*** We identified a novel, heterozygous, nonsense variant in *FOXA2* (NM_021784.5: c.C1260G; p.Y420X, Figure 2F) in a 24-year-old woman with a history of three spontaneous abortions, Heterozygous knockout mice for this gene had an abnormal miscarriage rate that mimicked the miscarriage phenotype in humans (Weinstein et al., 1994). Therefore, *FOXA2* has been prioritized as a strong candidate gene associated with human RPL.
(ii.) FGA. Two heterozygous *FGA* variants were identified in two women, each with three consecutive miscarriages. One variant (NM_000508.5: c.1906_1908del; p.636del, Figure 2B) leading to the deletion of an amino acid was not found in public databases. The other variant in *FGA* (NM_000508.5: c.C2285T; p.A762V; Figure 2C) causing an amino acid substitution was extremely rare in East Asian populations in the gnomAD database and was predicted to be deleterious by online programs. *FGA* encodes the alpha subunit of the coagulation factor fibrinogen. Mutations of *FGA* have been linked to coagulation pathologies including afibrinogenemia (OMIM:202400) and dysfibrinogenemia/hypodysfibrinogenemia (OMIM:616004), which can result in miscarriage (Valiton et al., 2019).
(iii.) F13A1. We identified two variants in *F13A1*, another blood coagulation-associated gene, in two patients. One 28-year-old woman with a history of four miscarriages had an extremely rare nonsense variant (NM_000129.4: c.C1201T; p.Q401X, Figure 2D) that resulted in a truncated protein. Another woman with a history of two miscarriages had a missense variant (NM_000129.4: c.C1834T; p.R612C, Figure 2E) that was predicted *in silico* to be deleterious. Recessive variants of *F13A1* cause F13 deficiency, a rare but severe hemorrhagic disorder featured by bleeding, delayed wound healing and spontaneous abortion (Karimi et al., 2009).
(iv.) KHDC3L. We identified a heterozygous in frame deletion in *KHDC3L* (NM_001017361.3: c.436_468del; p.146_156del; Figure 2A) in a 31-year-old woman with a history of two miscarriages. Maternal bi-allelic variants of *KHDC3L* are known to cause recurrent hydatidiform mole, an aberrant human pregnancy featuring early embryonic arrest and excessive trophoblastic proliferation (Nguyen et al., 2018), while heterozygous deletions (p.150_160del and p.150_172del) were found in patients experiencing RPL without forming an hydatidiform mole (Zhang et al., 2019). All of the deletions in patients with RPL affected the Thr156 residue, a critical phosphorylation site for normal KHDC3L protein function.
(v.) Other candidate genes. In addition to the six variants of causal genes aforementioned, we identified 29 variants of 24 candidate genes from the RPL-associated gene set in 26 patients (Table 2). There were 24 missense and one nonsense variants, three non-frameshift and one frameshift deletions. The DNA chromatograms of these variants are shown in Supplemental Figure S2. These genes are involved in several biological processes that could be relevant to maintaining a normal pregnancy, including angiogenesis, cell growth, immunity and inflammation response, and hormone signaling.

**TABLE 1:** Potential causative variants in patients with recurrent pregnancy loss.

Patient ID	Age, years	No. of miscarriage	Gene	Variant	Frequency^a^	ACMG/AMP	Relevance
53	24	3	FOXA2	NM_021784.5: c.C1260G; p.Y420X	0/0	LP	Function: maintaining the develop of endometrial glands. MGI phenotype: abnormal miscarriage rate
15	31	2	KHDC3L	NM_001017361.3: c.436_468del; p.146_156del	0.000032/0.00027	P	Function: keeping genetic stability of early embryonic cells. Human disease: hydatidiform mole, RPL. MGI phenotype: reduced female fertility.
4	29	3	FGA	NM_000508.5: c.1906_1908del; p.636del	0/0	US	Function: encoding alpha subunit of the coagulation factor fibrinogen. Human disease: afibrinogenemia, dysfibrinogenemia, hypodysfibrinogenemia. MGI phenotype: uterine hemorrhage, abnormal uterine environment, female infertility.
83	26	3	FGA	NM_000508.5: c.C2285T; p.A762V	0.00017/0.00005	US	
6	28	4	F13A1	NM_000129.4: c.C1201T; p.Q401X	0.000004/0.000054	P	Function: encoding the coagulation factor XIII A subunit. Human disease: factor XIIIA deficiency. MGI phenotype: uterine hemorrhage, reduced female fertility
37	30	2	F13A1	NM_000129.4: c.C1834T; p.R612C	0.000028/0.00025	US	

a Frequency in overall population/East Asian population in gnomAD.

P, pathogenic; LP, likely pathogenic; US, uncertain significance.

**TABLE 2:** The other heterozygous candidate variants identified by whole-exome sequencing in patients with recurrent pregnancy loss.

Patient ID	Gene	Variant	Frequency^a^	ACMG/AMP	Biological process relevant to pregnancy
1	ADAMTS1	NM_006988:c.G1811A:p.R604H	0.000004/0	US	Angiogenesis
54	NOS3	NM_000603:c.G1507A:p.V503M	0.000004/0	US	
48	S1PR3	NM_005226:c.38delG:p.R13fs	0/0	LP	
86	ASH1L	NM_018489:c.C7906T:p.P2636S	0/0	US	Chromatin modifying
89	ASH1L	NM_018489:c.C1411T:p.R471W	0.000008/0	US	
30	BIN1	NM_139343:c.C593T: p.T198I	0.00002/ 0.0002	US	Cell growth, proliferation, differentiation, apoptosis
62	LPAR3	NM_012152:c.G373A:p.V125M	0.00014/ 0.00033	US	
92	DDR1	NM_013993:c.C2404T:p.R802W	0/0	US	
22	PARL	NM_018622:c.C153G: p.C51W	0.000036/0.00049	US	
28	PARL	NM_018622:c.C153G: p.C51W	0.000036/0.00049	US	
9	SRC	NM_005417:c.C1337T:p.S446L	0.000004/0	US	
40	ROR2	NM_004560:c.T1612C:p.C538R	0/0	US	
31	ROR2	NM_004560:c.G1687A:p.E563K	0.000036/ 0.00005	US	
78	ARHGDIA	NM_001301243:c.357_374del:p.119_125del	0/0	US	
70	TNC	NM_002160:c.G434A:p.G145D	0.000004/0	US	Extracellular matrix
40	MMP10	NM_002425:c.G1168A:p.A390T	0/0	US	
76	MMP9	NM_004994:c.G473T: p.R158L	0/0	US	
65	C3	NM_000064:c.T1474C:p.Y492H	0/0	US	Immune and inflammation
74	C3	NM_000064:c.G3433A:p.A1145T	0.000019/ 0.00027	US	
27	NLRP2	NM_017852:c.C2342T:p.P781L	0/0	US	
45	OSBPL5	NM_020896:c.G1157A:p.R386H	0.000016/0	US	Chromosomal segregation
56	CENPB	NM_001810: c.1262_1264del:p.421_422del	0/0	US	
18	PER1	NM_002616:c.C278T: p.T93I	0.0000049/ 0.000058	US	Metabolism
67	SLC13A1	NM_022444:c.C814T: p.R272C	0.00022/ 0.00022	US	
34	TYR	NM_000372:c.C346T: p.R116X	0.000024/ 0.00016	P	
84	REXO4	NM_020385:c.192_206del:p.64_69del	0/0	US	Hormone signaling
45	REXO4	NM_020385:c.C976G: p.H326D	0/0	US	
78	FSHR	NM_000145:c.C491A: p.S164Y	0/0	LP	
50	FKBP4	NM_002014:c.C1066T:p.L356F	0.00008/ 0.00097	US	

a Frequency in overall population/East Asian population in gnomAD.

P, pathogenic; LP, likely pathogenic; US, uncertain significance.

### Effects of Knockdown of FOXA2 on Leukemia Inhibitory Factor Expression

**FIGURE 3:**
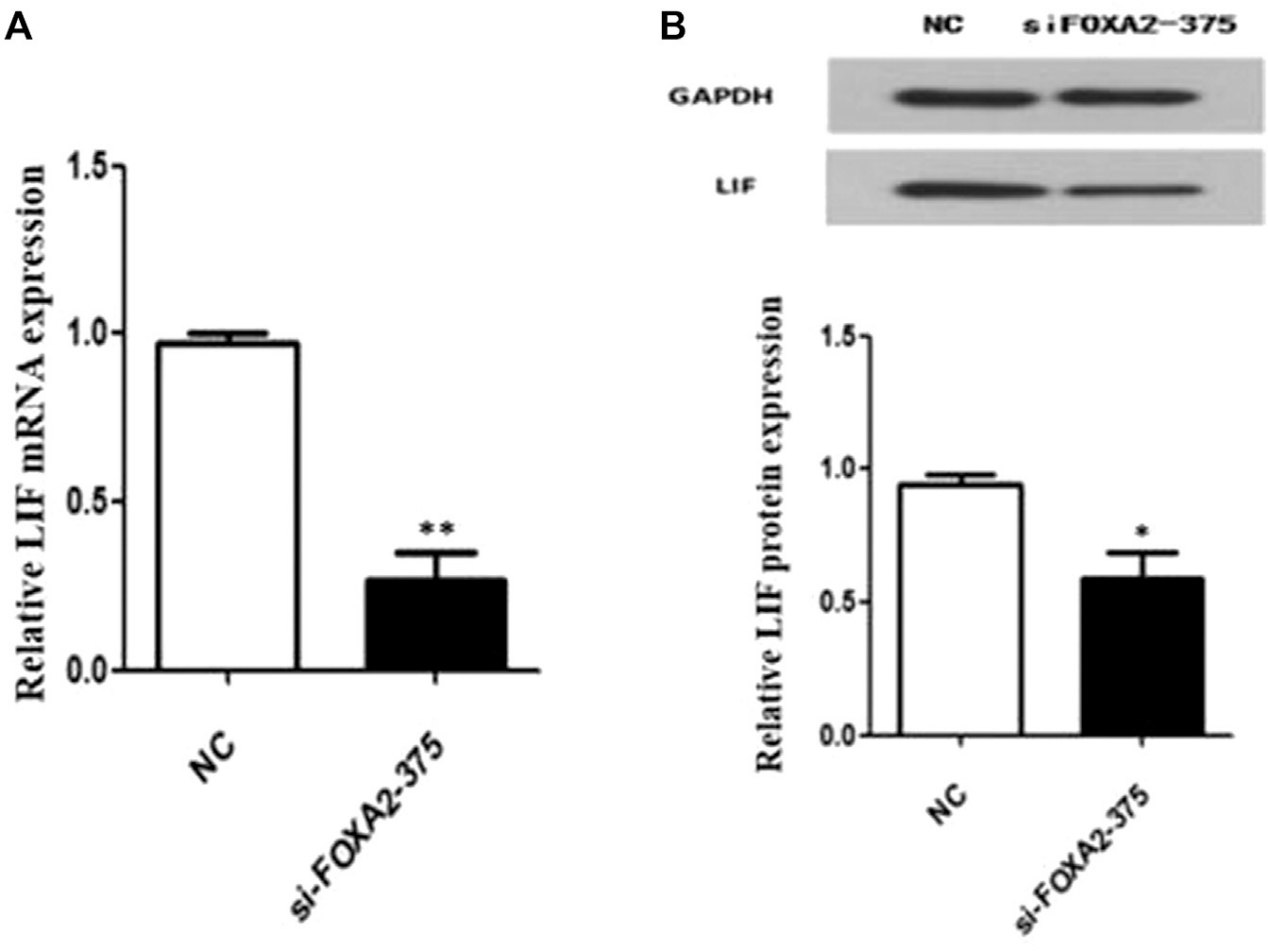
Expression of LIF in HEK 293T cells with FOXA2 knockdown. **(A)** The mRNA level of LIF was measured by qPCR. **(B)** The protein level of LIF was detected by western blot. Student’s t-test was used for the *p* value analysis. *p < 0.05; **p < 0.01.

To further explore the possible role of FOXA2 in pregnancy, the expression of a key embryo implantation marker—LIF, was detected after siFOXA2-375 transfection. The results of RT–qPCR and western blotting showed that both the mRNA and protein levels of LIF were significantly decreased in HEK-293T cells after downregulation of FOXA2 expression (Figure 3). Thus, knockdown of *FOXA2* might result in decreased LIF expression.

## Discussion

Here we performed WES on samples from 100 unrelated Han Chinese women, aiming to identify novel genes and variants associated with RPL. To more effectively identify candidate genes, we generated a subset of 95 RPL-associated genes including causative/candidate genes previously reported in patients with RPL and female infertility phenotype-associated genes identifies from mouse model studies. We identified six pathogenic or likely pathogenic variants in *FOXA2*, *FGA*, *F13A1,* and *KHDC3L,* which were considered potential causal genes according to the genetic findings, functional relevance and/or phenotypes of corresponding mouse models. We found 29 additional rare variants in the other candidate genes.

Pregnancy is an extremely complex physiological process that requires the participation of various hormones and cytokines. Previous studies on sheep and mice lacking uterine glands have provided direct evidence that these can contribute to the formation and continuation of pregnancy by secreting important components, e.g., LIF (Yamagami et al., 2014). *FOXA2* holds particular interest because it is expressed in endometrial glands and its expression increases transiently during early pregnancy in the rat (Yamagami et al., 2014). Previous findings provided clear evidence that *FOXA2* regulates the expression of LIF from uterine glands that is critical for blastocyst implantation and the development of uterine glands (Jeong et al., 2010; Kelleher et al., 2017). In addition, Lin *et al.* conducted a series of functional experiments to prove that *FOXA2* expression may affect the proliferation and migration of endometrium cells, leading to reproductive health diseases such as endometriosis. Furthermore, mice with a heterozygous knockout of FOXA2 had an elevated miscarriage rate because of abnormal blastocyst implantation and decidualization, similar to the phenotype of RPL. We found a newly identified heterozygous nonsense variant of *FOXA2* (c.C1260G; p.Y420X) in one patient. Therefore, we prioritized this *FOXA2* variant as a potential causative variant for RPL. This is the first *FOXA2* variant reported in cases of RPL.

LIF, highly expressed in the uterine endometrial glands in both mice and humans, plays an important role in maternal receptivity to blastocyst implantation, placental formation and in the development of the nervous system (Stewart et al., 1992; Charnock-Jones et al., 1994). Kelleher *et al.* (Kelleher et al., 2017) proved that LIF is not expressed during early pregnancy in adult *FOXA2*-knockout mice; moreover, injection of LIF could induce embryo implantation and rescue pregnancy. This finding using a mice model suggested that *FOXA2* might affect pregnancy by regulating the expression of LIF. Consistent with previous results, we found that knockdown of FOXA2 expression significantly decreased the expression of LIF in HEK-293T cells. Thus, *FOXA2* might be a candidate gene for variants causing RPL, by regulating the expression of LIF, a critical implantation factor of uterine gland origin. However, the specific pathways and mechanisms by which *FOXA2* variants lead to RPL clearly need further studies.

The complex balance of coagulability and hemorrhage from embryonic implantation to delivery are pivotal to the success of pregnancy. Abnormal coagulation can inhibit implantation and initiate miscarriage. In the past decades, several studies have suggested that polymorphisms of thrombophilic factors such as G1691A encoded by *FVL*, G20210A encoded by *F2* and C677T encoded by *MTHFR* have significant associations with the risk of RPL (Sergi et al., 2015; Chen et al., 2016; Jusic et al., 2018; Perés Wingeyer et al., 2019). Rare variants of blood coagulation-associated genes that distort the normal structure or function of the encoded proteins can disrupt the balance mentioned above, cause abnormal hemorrhage in the uterus during pregnancy and result in adverse pregnancy outcomes. Here we identified four variants in two coagulation-associated genes, *FGA* and *F13A1*, in four cases of RPL.

*FGA*, encoding the subunit of the coagulation factor fibrinogen, is an important component of maternal fibrinogen mainly involved in pregnancy by supporting the proliferation and diffusion of early trophoblast cells and by maintaining development of the fetomaternal circulation (Inbal and Muszbek 2003; Iwaki and Castellino 2005). Thus, *FGA* variants can result in disorders in fibrinogenemia (de Moerloose et al., 2013). For example, homozygous variants can cause afibrinogenemia a serious bleeding disorder (Robert-Ebadi et al., 2009; Amri et al., 2016), so pregnant women with afibrinogenemia are at greater risk of bleeding complications and RPL because of the absence of this key protein (Peyvandi et al., 2011). Heterozygous variants can lead to dysfibrinogenemia or hypofibrinogenemia (Casini et al., 2015), which are often clinically asymptomatic in most patients except in pregnant women who can develop significant bleeding for gynecological reasons (Castaman et al., 2019). Pregnant women with dysfibrinogenemia can suffer from obstetric complications including miscarriage mostly during the first trimester because of an abnormal decrease in fibrinogen levels and modifications of blood clot structures during pregnancy (Haverkate and Samama 1995). Li *et al.* (Li et al., 2018) reported four asymptomatic cases in women with congenital hypofibrinogenemia, one of whom had a history of six early miscarriages. Valiton *et al.* (Valiton et al., 2019) found that almost half of all pregnancies in women with hypofibrinogenemia and dysfibrinogenemia resulted in miscarriage. Here, we identified a novel in-frame deletion (NM_000508.5: c.1906_1908del; p.636del) and a rare missense variant (NM_000508.5: c.C2285T; p.A762V) in *FGA* in two patients who had both experienced three miscarriages. A rare *FGA* variant (c.T2054G; p.F685C) was also reported by Quintero-Ronderos *et al.* (Quintero-Ronderos et al., 2017a). All of these variants reside in the C-terminal region. Fragment molecular orbital analysis showed that the p.F685C variant led to changes in total interaction energy, thus leading to protein instability (Quintero-Ronderos et al., 2017a).

Another coagulation-associated gene, *F13A1*, encodes the A subunit of FXIII and is mainly expressed in the uterus and placenta (Shi and Wang 2017). *F13A1* homozygous variants might cause FXIII deficiency, and heterozygous missense variants will still have a strong effect on the functional status of the protein (Biswas et al., 2014; Thomas et al., 2016). Women with FXIII deficiency could suffer from detachment of the placenta from the uterus and subsequent miscarriage because of insufficient formation of the cytotrophoblastic shell and abnormal cross-linking of fibrin to fibronectin (Dorgalaleh and Rashidpanah 2016). A meta-analysis found that a common missense variant (p.V34L) was significantly associated with the risk of RPL in Asian women, indicating that functional variants in *F13A1* can be associated with this averse outcome (Jung et al., 2017). Our study identified two rare heterozygous variants of *F13A1* in two women who had experienced two and four miscarriages. One was a missense variant (c.C1834T; p.R612C), which is located in the Factor XIII-A barrel 1 region including an important Tyr560 residue crucial for the activation of FXIII-A zymogen (Thomas et al., 2016). The other was a nonsense variant (c.C1201T; p.Q401X) that causes a truncated protein and the loss of the protein’s core catalytic region. Our results, together with previous findings, provide evidence on the role of coagulation-associated genes (e.g., *FGA* and *F13A1*) in the etiology of RPL.

The KHDC3L protein is an important component of the subcortical maternal complex and is mainly found in oocytes. Rare biallelic variants of maternal *KHDC3L* have been reported to be associated with recurrent hydatidiform mole (Rezaei et al., 2016; Ji et al., 2019). Zhang *et al.* (Zhang et al., 2019) using a series of functional experiments found that *KHDC3L* could maintain the stability of the embryonic genome. Mechanically, two key residues, Thr145 and Thr156, phosphorylated by the Ataxia-telangiectasia mutated kinase, are critical for the functions of *KHDC3L*. Zhang *et al.* (Zhang et al., 2019) also detected two heterozygous in-frame deletions (p.150_160del and p.150_172del) in two of 29 patients with RPL. These two heterozygous deletions caused the loss of the critical residue Thr156 and led to abnormal instability of the embryonic genome and pregnancy failure by a dominant–negative effect (Zhang et al., 2019). Here, the heterozygous deletion (NM_001017361: c.436_468del) we found was consistent with a previously reported variant that also resulted in the loss of both phosphorylation sites. Our results further emphasize the pathogenic role of *KDHC3L* in RPL.

Regarding the other genes identified here, they are also of interest and might be candidates for RPL, because they are involved in biological processes that might be related to pregnancy loss: angiogenesis, the immune response, metabolism, extracellular matrix remodeling and regulation of critical cell functions. In addition to their functional relevance to human pregnancy and mouse model phenotypes, several genes have also been reported previously in patients with RPL with rare and potentially pathogenic variants. These include *MMP9*, *MMP10*, *TNC*, *FKBP4*, and *ATAMTS1*. However, the functional and phenotypic associations between these variants and RPL need further analysis. Our findings might provide some clues for future research on RPL. Furthermore, we found that there was no significant difference between patients with two miscarriages and those with three or four in age and variant accumulation, which is consistent with the view of some experts that the risk of having a second miscarriage in patients with two miscarriages is similar to that in those who had three miscarriages. This result further confirmed the importance of paying more attention and enhanced genetic counseling for patients who have experienced two consecutive miscarriages.

However, there were some limitations to our study. First, we did not study women with normal pregnancies and the lack of control samples may limit the interpretation of our results. Considering the high genetic heterogeneity of RPL and large numbers of genes involved in its development, it is difficult to reach statistical significance for case-control analyses with a limited sample size. Therefore, we focused on rare (i.e., frequency <0.1%), deleterious variants that were functionally relevant to miscarriage and used populations from public databases as controls. Further case-control studies with a large sample size based on whole exome sequencing are needed to more comprehensively unveil the genetic factors for RPL. Second, to explore more novel maternal-effect genes and variants in this study, we only sequenced samples of RPL patients seeking for maternal-effect genes but did not sequence samples from miscarried fetuses or from the husbands for fetal- and paternal-effect genes. The etiology of some RPL cases might have been missed in our study. Third, the pathogenic role of candidate variants identified in our study clearly needs verification by functional experiments in the future.

In summary, we found rare variants in known causative genes and identified some newly identified candidate genes for RPL by WES analysis in 100 unrelated Han Chinese women with RPL. The detection of several variants in coagulation-associated genes further emphasizes the importance of a blood coagulation balance in pregnancy and variant screening of coagulation-associated genes might be useful in patients with RPL even if they are asymptomatic. We identified *FOXA2* as a new candidate gene with variants causing RPL, and a subset of genes that might be associated with miscarriage. The findings might provide some genetic clues for future functional research and clinical intervention. Therefore, large-scale, next-generation sequencing studies for RPL and functional investigations for candidate genes are clearly needed in the future.

## Data Availability

The original contributions presented in the study are included in the article/Supplementary Material, further inquiries can be directed to the corresponding authors.

## References

[B1] Amri Y. Toumi N. E. H. Hadj Fredj S. De Moerloose P. (2016). Congenital Afibrinogenemia: Identification and Characterization of Two Novel Homozygous Fibrinogen Aα and Bβ Chain Mutations in Two Tunisian Families. Thromb. Res. 143, 11–16. 10.1016/j.thromres.2016.04.016 27164460

[B2] Arias-Sosa L. A. Acosta I. D. Lucena-Quevedo E. Moreno-Ortiz H. Esteban-Pérez C. Forero-Castro M. (2018). Genetic and Epigenetic Variations Associated with Idiopathic Recurrent Pregnancy Loss. J. Assist. Reprod. Genet. 35, 355–366. 10.1007/s10815-017-1108-y 29313278 PMC5904072

[B3] Biswas A. Ivaskevicius V. Thomas A. Varvenne M. Brand B. Rott H. (2014). Eight Novel F13a1 Gene Missense Mutations in Patients with Mild Fxiii Deficiency: In Silico Analysis Suggests Changes in Fxiii-A Subunit Structure/function. Ann. Hematol. 93, 1665–1676. 10.1007/s00277-014-2102-4 24889649

[B4] Casini A. Neerman-Arbez M. Ariëns R. A. De Moerloose P. (2015). Dysfibrinogenemia: From Molecular Anomalies to Clinical Manifestations and Management. J. Thromb. Haemost. 13, 909–919. 10.1111/jth.12916 25816717

[B5] Casini A. Neerman-Arbez M. de Moerloose P. (2013). Congenital Fibrinogen Disorders: An Update. Semin. Thromb. Hemost. 39, 585–595. 10.1055/s-0033-1349222 23852822

[B6] Castaman G. Giacomelli S. H. Biasoli C. Contino L. Radossi P. (2019). Risk of Bleeding and Thrombosis in Inherited Qualitative Fibrinogen Disorders. Eur. J. Haematol. 103, 379–384. 10.1111/ejh.13296 31314131

[B7] Charnock-Jones D. S. Sharkey A. M. Fenwick P. Smith S. K. (1994). Leukaemia Inhibitory Factor Mrna Concentration Peaks in Human Endometrium at the Time of Implantation and the Blastocyst Contains Mrna for the Receptor at This Time. Reproduction 101, 421–426. 10.1530/jrf.0.1010421 7932378

[B8] Chen H. Yang X. Lu M. (2016). Methylenetetrahydrofolate Reductase Gene Polymorphisms and Recurrent Pregnancy Loss in china: A Systematic Review and Meta-Analysis. Arch. Gynecol. Obstet. 293, 283–290. 10.1007/s00404-015-3894-8 26399758

[B9] Demetriou C. Chanudet E. JosephJoseph A. Topf M. Thomas A. C. Bitner-Glindzicz M. (2019). Exome Sequencing Identifies Variants in Fkbp4 that Are Associated with Recurrent Fetal Loss in Humans. Hum. Mol. Genet. 28, 3466–3474. 10.1093/hmg/ddz203 31504499

[B10] Dorgalaleh A. Rashidpanah J. (2016). Blood Coagulation Factor Xiii and Factor Xiii Deficiency. Blood Rev. 30, 461–475. 10.1016/j.blre.2016.06.002 27344554

[B11] El Hachem H. Crepaux V. May-Panloup P. Descamps P. Legendre G. Bouet P.-E. (2017). Recurrent Pregnancy Loss: Current Perspectives. Ijwh Vol. 9, 331–345. 10.2147/IJWH.S100817 PMC5440030 28553146

[B12] Haverkate F. Samama M. (1995). Familial Dysfibrinogenemia and Thrombophilia. Thromb. Haemost. 73, 151–161. 10.1055/s-0038-1653741 7740487

[B13] Inbal A. Muszbek L. (2003). Coagulation Factor Deficiencies and Pregnancy Loss. Semin. Thromb. Hemost. 29, 171–174. 10.1055/s-2003-38832 12709920

[B14] Iwaki T. Castellino F. (2005). Maternal Fibrinogen Is Necessary for Embryonic Development. Cdt 6, 535–539. 10.2174/1389450054546006 16026273

[B15] Jeong J.-W. Kwak I. Lee K. Y. Kim T. H. Large M. J. Stewart C. L. (2010). Foxa2 Is Essential for Mouse Endometrial Gland Development and Fertility1. Biol. Reprod. 83, 396–403. 10.1095/biolreprod.109.083154 20484741 PMC2924802

[B16] Ji M. Shi X. Xiang Y. Cui Q. Zhao J. (2019). Nlrp7 and Khdc3l Variants in Chinese Patients with Recurrent Hydatidiform Moles. Jpn. J. Clin. Oncol. 49, 620–627. 10.1093/jjco/hyz036 31220306

[B17] Jung J. H. Kim J.-H. Song G. G. Choi S. J. (2017). Association of the F13a1 Val34leu Polymorphism and Recurrent Pregnancy Loss: A Meta-Analysis. Eur. J. Obstet. Gynecol. Reprod. Biol. 215, 234–240. 10.1016/j.ejogrb.2017.06.032 28683377

[B18] Jusic A. Balic D. Avdic A. Podanin M. Balic A. (2018). The Association of Factor V G1961a (Factor V Leiden), Prothrombin G20210a, Mthfr C677t and Pai-1 4g/5g Polymorphisms with Recurrent Pregnancy Loss in Bosnian Women. Med. Glas (Zenica) 15, 158–163. 10.17392/948-18 29703881

[B19] Karimi M. Bereczky Z. Cohan N. Muszbek L. (2009). Factor Xiii Deficiency. Semin. Thromb. Hemost. 35, 426–438. 10.1055/s-0029-1225765 19598071

[B20] Kelleher A. M. Peng W. Pru J. K. Pru C. A. Demayo F. J. Spencer T. E. (2017). Forkhead Box A2 (Foxa2) Is Essential for Uterine Function and Fertility. Proc. Natl. Acad. Sci. USA 114, E1018–E1026. 10.1073/pnas.1618433114 28049832 PMC5307455

[B21] Kolte A. M. Nielsen H. S. Moltke I. Degn B. Pedersen B. Sunde L. (2011). A Genome-wide Scan in Affected Sibling Pairs with Idiopathic Recurrent Miscarriage Suggests Genetic Linkage. Mol. Hum. Reprod. 17, 379–385. 10.1093/molehr/gar003 21257601

[B22] Li C. Q. Wang D. X. Wei X. Y. (2018). Perioperative Management of Pregnant Women Combined with Congenital Fibrinogen Deficiency: Four Cases Report and Literature Review. Beijing Da Xue Xue Bao Yi Xue Ban 50, 932–936. 30337762

[B23] Li Wang W. Zeng Chan Wang W. Cui Xie X. Xiao Feng Liu L. Mao Sheng Yang Y. (2010). Genome-wide Screening for Risk Loci of Idiopathic Recurrent Miscarriage in a Han Chinese Population: A Pilot Study. Reprod. Sci. 17, 578–584. 10.1177/1933719110364248 20305137

[B24] Maddirevula S. Awartani K. Coskun S. Alnaim L. F. Ibrahim N. Abdulwahab F. (2020). A Genomics Approach to Females with Infertility and Recurrent Pregnancy Loss. Hum. Genet. 139, 605–613. 10.1007/s00439-020-02143-5 32172300

[B25] Mohlin F. C. Gros P. Mercier E. Gris J.-C. R. Blom A. M. (2018). Analysis of C3 Gene Variants in Patients with Idiopathic Recurrent Spontaneous Pregnancy Loss. Front. Immunol. 9, 1813. 10.3389/fimmu.2018.01813 30131807 PMC6090058

[B26] Mohlin F. C. Mercier E. Fremeaux-Bacchi V. Liszewski M. K. Atkinson J. P. Gris J.-C. (2013). Analysis of Genes Coding for Cd46, Cd55, and C4b-Binding Protein in Patients with Idiopathic, Recurrent, Spontaneous Pregnancy Loss. Eur. J. Immunol. 43, 1617–1629. 10.1002/eji.201243196 23508668 PMC3760018

[B27] Nguyen N. M. P. Khawajkie Y. Mechtouf N. Rezaei M. Breguet M. Kurvinen E. (2018). The Genetics of Recurrent Hydatidiform Moles: New Insights and Lessons from a Comprehensive Analysis of 113 Patients. Mod. Pathol. 31, 1116–1130. 10.1038/s41379-018-0031-9 29463882

[B28] Page J. M. Silver R. M. (2016). Genetic Causes of Recurrent Pregnancy Loss. Clin. Obstet. Gynecol. 59, 498–508. 10.1097/GRF.0000000000000217 27414972

[B29] Pan H. Xiang H. Wang J. Wei Z. Zhou Y. Liu B. (2019). Caps Mutations Are Potentially Associated with Unexplained Recurrent Pregnancy Loss. Am. J. Pathol. 189, 124–131. 10.1016/j.ajpath.2018.09.010 30339840

[B30] Perés Wingeyer S. Aranda F. Udry S. Latino J. de Larrañaga G. (2019). Inherited Thrombophilia and Pregnancy Loss. Study of an Argentinian Cohort. Medicina Clínica (English Edition) 152, 249–254. 10.1016/j.medcli.2017.12.019 29523337

[B31] Pereza N. Ostojić S. Kapović M. Peterlin B. (2017). Systematic Review and Meta-Analysis of Genetic Association Studies in Idiopathic Recurrent Spontaneous Abortion. Fertil. Sterility 107, 150–159. 10.1016/j.fertnstert.2016.10.007 27842992

[B32] Peyvandi F. Bidlingmaier C. Garagiola I. (2011). Management of Pregnancy and Delivery in Women with Inherited Bleeding Disorders. Semin. Fetal Neonatal Med. 16, 311–317. 10.1016/j.siny.2011.07.006 21852211

[B33] Practice Committee of the American Society for Reproductive Medicine (2020). Definitions of Infertility and Recurrent Pregnancy Loss: a Committee Opinion. Fertil. Sterility 113, 533–535. 10.1016/j.fertnstert.2019.11.025 32115183

[B34] Practice Committee of the American Society for Reproductive, M. (2012). Evaluation and Treatment of Recurrent Pregnancy Loss: A Committee Opinion. Fertil. Sterility 98, 1103–1111. 10.1016/j.fertnstert.2012.06.048 22835448

[B35] Qian J. Nguyen N. M. P. Rezaei M. Huang B. Tao Y. Zhang X. (2018). Biallelic Padi6 Variants Linking Infertility, Miscarriages, and Hydatidiform Moles. Eur. J. Hum. Genet. 26, 1007–1013. 10.1038/s41431-018-0141-3 29693651 PMC6018785

[B36] Qiao Y. Wen J. Tang F. Martell S. Shomer N. Leung P. C. K. (2016). Whole Exome Sequencing in Recurrent Early Pregnancy Loss. Mol. Hum. Reprod. 22, 364–372. 10.1093/molehr/gaw008 26826164 PMC4847612

[B37] Quintero-Ronderos P. Jiménez K. M. Esteban-Pérez C. Ojeda D. A. Bello S. Fonseca D. J. (2019). Foxd1 Mutations Are Related to Repeated Implantation Failure, Intra-uterine Growth Restriction and Preeclampsia. Mol. Med. 25, 37. 10.1186/s10020-019-0104-3 31395028 PMC6688323

[B38] Quintero-Ronderos P. Laissue P. (2019). Genetic Variants Contributing to Early Recurrent Pregnancy Loss Etiology Identified by Sequencing Approaches. Reprod. Sci. 1933719119831769, 193371911983176. 10.1177/1933719119831769 30879428

[B39] Quintero-Ronderos P. Mercier E. Fukuda M. González R. Suárez C. F. Patarroyo M. A. (2017a). Novel Genes and Mutations in Patients Affected by Recurrent Pregnancy Loss. PLoS One. 12:e0186149. 10.1371/journal.pone.0186149 29016666 PMC5634651

[B40] Quintero-Ronderos P. Mercier E. Gris J.-C. Esteban-Perez C. Moreno-Ortiz H. Fonseca D. J. (2017b). Thbd Sequence Variants Potentially Related to Recurrent Pregnancy Loss. Reprod. Biol. Endocrinol. 15, 92. 10.1186/s12958-017-0311-0 29195508 PMC5709961

[B41] Reichert S. L. Mckay E. M. Moldenhauer J. S. (2016). Identification of a Novel Nonsense Mutation in theFOXP3gene in a Fetus with Hydrops-Expanding the Phenotype of IPEX Syndrome. Am. J. Med. Genet. 170, 226–232. 10.1002/ajmg.a.37401 26395338

[B42] Rezaei M. Nguyen N. M. P. Foroughinia L. Dash P. Ahmadpour F. Verma I. C. (2016). Two Novel Mutations in the Khdc3l Gene in Asian Patients with Recurrent Hydatidiform Mole. Hum. Genome 3, 16027. 10.1038/hgv.2016.27 PMC5007383 27621838

[B43] Robbins S. M. Thimm M. A. Valle D. Jelin A. C. (2019). Genetic Diagnosis in First or Second Trimester Pregnancy Loss Using Exome Sequencing: A Systematic Review of Human Essential Genes. J. Assist. Reprod. Genet. 36, 1539–1548. 10.1007/s10815-019-01499-6 31273585 PMC6707996

[B44] Robert-Ebadi H. De Moerloose P. Khorassani M. E. Khattab M. E. Neerman-Arbez M. (2009). A Novel Frameshift Mutation in Fga Accounting for Congenital Afibrinogenemia Predicted to Encode an Aberrant Peptide Terminating 158 Amino Acids Downstream. Blood Coagul. Fibrinolysis 20, 385–387. 10.1097/MBC.0b013e328329f2a0 19417632

[B45] Saravelos S. H. Regan L. (2014). Unexplained Recurrent Pregnancy Loss. Obstet. Gynecol. Clin. North America 41, 157–166. 10.1016/j.ogc.2013.10.008 24491990

[B46] Sergi C. Al Jishi T. Walker M. (2015). Factor V Leiden Mutation in Women with Early Recurrent Pregnancy Loss: A Meta-Analysis and Systematic Review of the Causal Association. Arch. Gynecol. Obstet. 291, 671–679. 10.1007/s00404-014-3443-x 25193429

[B47] Shamseldin H. E. Swaid A. Alkuraya F. S. (2013). Lifting the Lid on Unborn Lethal Mendelian Phenotypes through Exome Sequencing. Genet. Med. 15, 307–309. 10.1038/gim.2012.130 23037934 PMC3908556

[B48] Shi D. Y. Wang S. J. (2017). Advances of Coagulation Factor Xiii. Chin. Med. J. (Engl) 130, 219–223. 10.4103/0366-6999.198007 28091415 PMC5282680

[B49] Stewart C. L. Kaspar P. Brunet L. J. Bhatt H. Gadi I. Köntgen F. (1992). Blastocyst Implantation Depends on Maternal Expression of Leukaemia Inhibitory Factor. Nature 359, 76–79. 10.1038/359076a0 1522892

[B50] Thomas A. Biswas A. Dodt J. Philippou H. Hethershaw E. Ensikat H. J. (2016). Coagulation Factor Xiiia Subunit Missense Mutations Affect Structure and Function at the Various Steps of Factor Xiii Action. Hum. Mutat. 37, 1030–1041. 10.1002/humu.23041 27363989

[B51] Valiton V. Hugon‐Rodin J. Fontana P. Neerman‐Arbez M. Casini A. (2019). Obstetrical and Postpartum Complications in Women with Hereditary Fibrinogen Disorders: A Systematic Literature Review. Haemophilia 25, 747–754. 10.1111/hae.13825 31368232

[B52] Weinstein D. C. Ruiz I Altaba A. Chen W. S. Hoodless P. Prezioso V. R. Jessell T. M. (1994). The Winged-helix Transcription Factor HNF-3β Is Required for Notochord Development in the Mouse Embryo. Cell 78, 575–588. 10.1016/0092-8674(94)90523-1 8069910

[B53] Yamagami K. Yamauchi N. Kubota K. Nishimura S. Chowdhury V. S. Yamanaka K. (2014). Expression and Regulation of Foxa2 in the Rat Uterus during Early Pregnancy. J. Reprod. Dev. 60, 468–475. 10.1262/jrd.2014-086 25262775 PMC4284322

[B54] Yang Y. Luo Y. Yuan J. Tang Y. Xiong L. Xu M. (2016). Association between Maternal, Fetal and Paternal Mthfr Gene C677t and A1298c Polymorphisms and Risk of Recurrent Pregnancy Loss: A Comprehensive Evaluation. Arch. Gynecol. Obstet. 293, 1197–1211. 10.1007/s00404-015-3944-2 26530235

[B55] Zhang W. Chen Z. Zhang D. Zhao B. Liu L. Xie Z. (2019). Khdc3l Mutation Causes Recurrent Pregnancy Loss by Inducing Genomic Instability of Human Early Embryonic Cells. Plos Biol. 17, e3000468. 10.1371/journal.pbio.3000468 31609975 PMC6812846

[B56] Zhang Y. Li G. Fan Y. Cui Y. Huang S. Ma J. (2015). Novel Missense Mutation in Wnt6 in 100 Couples with Unexplained Recurrent Miscarriage. Hum. Reprod. 30, 994–999. 10.1093/humrep/dev028 25750203

