# Supplementary material for: Exome-Sequencing Identifies Novel Genes Associated with Recurrent Pregnancy Loss in a Chinese Cohort
Source: Front Genet. 2021 Dec 2;12:746082. doi: 10.3389/fgene.2021.746082 (PMC8674582; doi:10.3389/fgene.2021.746082)
Supplement: Supplementary file 1 [file Table1.DOCX]

Supplementary Table 1 RPL-related genes.

| Gene | Full name | MGI Phenotype | Transcript |
| --- | --- | --- | --- |
| ABCA1 | ATP-binding cassette, sub-family A (ABC1), member 1 | Uterus inflammation | NM_005502.4 |
| ADAMTS1 | ADAM metallopeptidase with thrombospondin type 1 motif 1 | Impaired embryo implantation | NM_006988.5 |
| ADCYAP1 | Adenylate cyclase activating polypeptide 1 | Impaired embryo implantation | NM_001099733.1 |
| AMHR2 | Anti-Mullerian hormone receptor type 2 | Failure of embryo implantation;  impaired embryo implantation;  abnormal uterine environment; abnormal miscarriage rate | NG_015981.1 |
| ANTXR2 | Anthrax toxin receptor 2 | Abnormal miscarriage rate; | NM_001145794.1 |
| ARHGDIA | Rho GDP dissociation inhibitor alpha | Abnormal uterine environment | [NM_001185077.3](https://www.ncbi.nlm.nih.gov/nuccore/NM_001185077.3) |
| ARNTL | Aryl hydrocarbon receptor nuclear translocator like | Failure of embryo implantation | [NM_001030272.2](https://www.ncbi.nlm.nih.gov/nuccore/NM_001030272.2) |
| ASH1L | ASH1 like histone lysine methyltransferase | Abnormal decidualization  abnormal post-implantation uterine environment | [NM_001366177.2](https://www.ncbi.nlm.nih.gov/nuccore/NM_001366177.2) |
| BIN1 | Bridging integrator 1 | Endometrium inflammation | NM_001320632.2 |
| BMP7 | Bone morphogenetic protein 7 | Decreased fetal size | NM_001719.2 |
| C3 | Complement C3 | Impaired embryo implantation | [NM_000064.4](https://www.ncbi.nlm.nih.gov/nuccore/NM_000064.4) |
| C4BPA | Complement component 4 binding protein alpha |  | NM_000715.4 |
| CAPS | Calcyphosine |  | NM_004058.5 |
| CBS | Cystathionine-beta-synthase | Abnormal uterine environment | NM_000071.2 |
| CBX3 | Chromobox 3 | Infertility | NM_007276.5 |
| CCDC68 | Coiled-coil domain containing 68 |  | NM_001143829.1 |
| CD46 | CD46 molecule | Abnormal fertilization | NM_002389.4 |
| CDH11 | Cadherin 11 | Incomplete somite formation | NM_001308392.2 |
| CDKN1C | Cyclin dependent kinase inhibitor 1C | Abnormal uterine environment | NM_000076.2 |
| CENPB | Centromere protein B | Abnormal uterine environment | [NM_001810.6](https://www.ncbi.nlm.nih.gov/nuccore/NM_001810.6) |
| CENPH | Centromere protein H |  | NM_022909.4 |
| COL6A3 | Collagen type VI alpha 3 chain |  | NM_004369.4 |
| CR1 | Complement C3b/C4b receptor 1 (Knops blood group) |  | NM_000573.4 |
| CSF1 | Colony stimulating factor 1 | Impaired embryo implantation | NG_030008.1 |
| CSF1R | Colony stimulating factor 1 receptor |  | NM_001288705.3 |
| DDR1 | Discoidin domain receptor family, member 1 | Failure of embryo implantation | [NM_001202521.1](https://www.ncbi.nlm.nih.gov/nuccore/NM_001202521.1) |
| DLGAP5 | DLG associated protein 5 | Abnormal decidualization | [NM_001146015.2](https://www.ncbi.nlm.nih.gov/nuccore/NM_001146015.2) |
| DNAH11 | Dynein axonemal heavy chain 11 | Abnormal embryo implantation | [NM_001277115.2](https://www.ncbi.nlm.nih.gov/nuccore/NM_001277115.2) |
| EPHA1 | EPH receptor A1 | Endometrium inflammation | [NM_005232.5](https://www.ncbi.nlm.nih.gov/nuccore/NM_005232.5) |
| ESR1 | Estrogen receptor 1 | Failure of embryo implantation | NM_001122742.1 |
| F13A1 | Coagulation factor XIII A chain | Uterine hemorrhage | [NM_000129.4](https://www.ncbi.nlm.nih.gov/nuccore/NM_000129.4) |
| FCER1G | Fc fragment of IgE receptor Ig | Abnormal uterine environment | [NM_004106.2](https://www.ncbi.nlm.nih.gov/nuccore/NM_004106.2) |
| FGA | Fibrinogen alpha chain | Abnormal uterine environment; uterine hemorrhage | NM_000508.3 |
| FGG | Fibrinogen gamma chain | Abnormal miscarriage rate; uterine hemorrhage | NM_021870.2 |
| FKBP4 | FK506 binding protein 4 | Failure of embryo implantation;  abnormal uterine receptivity | NM_002014.3 |
| FOXA2 | Forkhead box A2 | Abnormal miscarriage rate | [NM_021784.5](https://www.ncbi.nlm.nih.gov/nuccore/NM_021784.5) |
| FOXD1 | Forkhead box D1 |  | NM_004472.2 |
| FSHR | Follicle stimulating hormone receptor | Abnormal uterine environment; uterine hemorrhage | [NM_000145.4](https://www.ncbi.nlm.nih.gov/nuccore/NM_000145.4) |
| FUT2 | Fucosyltransferase 2 | Abnormal uterine environment | [NM_000511.6](https://www.ncbi.nlm.nih.gov/nuccore/NM_000511.6) |
| FZD4 | Frizzled class receptor 4 | Failure of embryo implantation | NM_012193.3 |
| GHR | Growth hormone receptor | Impaired embryo implantation | NG_011688.2 |
| HMX3 | H6 family homeobox 3 | Failure of embryo implantation;  abnormal decidualization | NM_001105574.1 |
| HORMAD1 | HORMA domain containing 1 | Abnormal preimplantation embryo development;  failure of embryo implantation;  abnormal female meiosis | [NM_001199829.2](https://www.ncbi.nlm.nih.gov/nuccore/NM_001199829.2) |
| HOXA11 | Homeobox A11 | Abnormal uterine environment | [NM_005523.6](https://www.ncbi.nlm.nih.gov/nuccore/NM_005523.6) |
| IDO2 | Indoleamine 2,3-dioxygenase 2 |  | NM_194294.2 |
| IL15 | Interleukin 15 | Abnormal decidualization  absent uterine NK cells | [NM_000585.5](https://www.ncbi.nlm.nih.gov/nuccore/NM_000585.5) |
| IL6ST | Interleukin 6 signal transducer | Failure of embryo implantation | [NM_001190981.2](https://www.ncbi.nlm.nih.gov/nuccore/NM_001190981.2) |
| ITGB3 | Integrin, beta 3 | Abnormal miscarriage rate; abnormal uterine environment; uterine hemorrhage | NM_000212.2 |
| KDM3B | Lysine demethylase 3B | Impaired embryo implantation  abnormal decidualization | [NM_016604.4](https://www.ncbi.nlm.nih.gov/nuccore/NM_016604.4) |
| KHDC3L | KH domain containing 3 like | Reduced female fertility | [NM_001017361.3](https://www.ncbi.nlm.nih.gov/nuccore/NM_001017361.3) |
| LIF | Leukemia inhibitory factor | Abnormal embryo attachment; failure of embryo implantation;  abnormal uterine receptivity | NM_002309.4 |
| LPAR3 | Lysophosphatidic acid receptor 3 | Impaired embryo implantation | [NM_012152.3](https://www.ncbi.nlm.nih.gov/nuccore/NM_012152.3) |
| MECOM | MDS1 and EVI1 complex locus | Abnormal miscarriage rate | [NM_001105077.3](https://www.ncbi.nlm.nih.gov/nuccore/NM_001105077.3) |
| METRNL | Meteorin like, glial cell differentiation regulator | Uterus inflammation | [NM_001004431.3](https://www.ncbi.nlm.nih.gov/nuccore/NM_001004431.3) |
| MMP10 | Matrix metallopeptidase 10 |  | NM_002425.3 |
| MMP9 | Matrix metallopeptidase 9 | Reduced fertility | NM_004994.3 |
| MRC1 | Mannose receptor, C type 1 | Impaired embryo implantation | NM_002438.4 |
| NCOA1 | Nuclear Receptor Coactivator 1 | Abnormal decidualization | NM_003743.4 |
| NCOA2 | Nuclear receptor coactivator 2 | Failure of embryo implantation | [NM_001321703.2](https://www.ncbi.nlm.nih.gov/nuccore/NM_001321703.2) |
| NCOA6 | Nuclear Receptor Coactivator 6 | Abnormal decidualization  abnormal uterine receptivity | [NM_001242539.2](https://www.ncbi.nlm.nih.gov/nuccore/NM_001242539.2) |
| NLRP10 | NLR family pyrin domain containing 10 |  | NM_176821.4 |
| NLRP2 | NLR family pyrin domain containing 2 |  | NM_001174081.3 |
| NLRP5 | NLR family pyrin domain containing 5 |  | NM_153447.4 |
| NLRP7 | NLR family pyrin domain containing 7 |  | NM_001127255.1 |
| NOS3 | Nitric oxide synthase 3 | Impaired embryo implantation | NM_000603.4 |
| OSBPL5 | Oxysterol binding protein like 5 |  | NM_001144063.2 |
| PADI6 | Peptidyl arginine deiminase 6 | Failure of zygotic cell division;  female infertility | NM_207421.4 |
| PARL | Presenilin associated rhomboid like | Abnormal uterine environment | [NM_001037639.3](https://www.ncbi.nlm.nih.gov/nuccore/NM_001037639.3) |
| PER1 | Period circadian clock 1 | Impaired embryo implantation | NM_002616.2 |
| PGR | Progesterone receptor | Uterus inflammation; abnormal decidualization | NM_000926.4 |
| PIF1 | PIF1 5'-to-3' DNA helicase |  | NM_001286496.2 |
| PLCD4 | Phospholipase C delta 4 |  | NM_032726.4 |
| PRDM1 | PR domain containing 1 | Uterine hemorrhage | [NM_001198.4](https://www.ncbi.nlm.nih.gov/nuccore/NM_001198.4) |
| PRL | Prolactin | Failure of embryo implantation | [NM_000948.6](https://www.ncbi.nlm.nih.gov/nuccore/NM_000948.6) |
| PRLR | Prolactin receptor | Abnormal uterine environment | NM_000949.5 |
| PTGS1 | Prostaglandin-endoperoxide synthase 1 | Abnormal uterine environment | NM_000962.2 |
| PTGS2 | Prostaglandin-endoperoxide synthase 2 | Failure of embryo implantation;  abnormal decidualization | [NM_000963.4](https://www.ncbi.nlm.nih.gov/nuccore/NM_000963.4) |
| REXO4 | REX4 homolog, 3'-5' exonuclease |  | NM_001279349.1 |
| ROR1 | Receptor tyrosine kinase-like orphan receptor 1 | Abnormal embryo attachment | NM_001083592.2 |
| ROR2 | Receptor tyrosine kinase-like orphan receptor 2 | Abnormal embryo attachment | NM_001318204.2 |
| S1PR3 | Sphingosine-1-phosphate receptor 3 | Abnormal miscarriage rate | [NM_005226.4](https://www.ncbi.nlm.nih.gov/nuccore/NM_005226.4) |
| SLC13A1 | Solute carrier family 13 member 1 | Abnormal miscarriage rate | [NM_001324400.1](https://www.ncbi.nlm.nih.gov/nuccore/NM_001324400.1) |
| SRC | V-src avian sarcoma (Schmidt-Ruppin A-2) viral oncogene homolog | Abnormal uterine environment | NM_005417.3 |
| STAT5B | Signal transducer and activator of transcription 5B | Abnormal miscarriage rate | NM_012448.3 |
| SWSAP1 | SWIM-type zinc finger 7 associated protein 1 | Impaired embryo implantation | [NM_175871.4](https://www.ncbi.nlm.nih.gov/nuccore/NM_175871.4) |
| SYCP3 | synaptonemal complex protein 3 | Abnormal female meiosis | NM_153694.4 |
| TLE6 | TLE family member 6, subcortical maternal complex member | Failure of zygotic cell division,  abnormal mitotic spindle morphology; female infertility | NM_001143986.2 |
| TLR3 | Toll-like receptor 3 | Abnormal post-implantation uterine environment | NM_003265.2 |
| TNC | Tenascin C |  | NM_002160.4 |
| TSHR | Thyroid stimulating hormone receptor | Abnormal uterine environment | [NM_000369.5](https://www.ncbi.nlm.nih.gov/nuccore/NM_000369.5) |
| TYR | Tyrosinase | Abnormal miscarriage rate | [NM_000372.5](https://www.ncbi.nlm.nih.gov/nuccore/NM_000372.5) |
| TYROBP | TYRO protein tyrosine kinase binding protein | Failure of embryo implantation;  abnormal maternal decidual layer morphology | NM_003332.3 |
| WNT5A | Wingless-type MMTV integration site family, member 5a | Abnormal embryo attachment | NM_003392.4 |
| WNT6 | Wingless-type MMTV integration site family, member 6 | Abnormal decidualization  abnormal embryo implantation; abnormal maternal decidual layer morphology | NM_006522.3 |
| WNT7A | Wingless-type MMTV integration site family, member 7A | Abnormal post-implantation uterine environment | NM_004625.3 |

Supplementary Table 2 Primers for Sanger sequencing

| Gene | Variant | Primer | Length, bp |
| --- | --- | --- | --- |
| KHDC3L | c.436_468del | F: GGGGCTGGGAATAGGGCTAC  R: TTCTGGGCGGAAATACGG | 650 |
| FGA | c.1906_1908del | F: CCGAAGATGGTTCTGACT  R: AGCGGTTATTTATACCTACA | 965 |
|  | c.C2285T | F: GGCAGCCTGAATGACGAG  R: AAAGGAAACCCAGACCAC | 450 |
| F13A1 | c.C1201T | F: GCTCACTGTAACCTCCATA  R: AGTTAGAAGACAAATACCCAC | 889 |
|  | c.C1834T | F: TCCAGCACATTCTTTACC  R: ACTATCCACAATTATACCTCC | 787 |
| FOXA2 | c.C1260A | F: AGAACTCCATCCGCCACTC  R: GGTCATTTACAACAGACCCTTACA | 1262 |
| ADAMTS1 | c.G1811A | F:AAGCCTCAGAATCCCATAC | 1254 |
|  |  | R:TGAAGCCTAGTCGCACAG |  |
| NOS3 | c.G1507A | F: AAGAATGGGCGAGGTCTGT | 1081 |
|  |  | R: ACCAGCGTCTCGTGTTCG |  |
| S1PR3 | c.38delG | F: ATCAATGGTCGCTCCCTC | 635 |
|  |  | R: CCTCTTGTTGGCGTCGTAA |  |
| ASH1L | c.C7906T | F:ATTAAGTGGGTTCTGTGC | 1149 |
|  |  | R: AATGCGAAAGATGTCAAG |  |
|  | c.C1411T | F: TAAACCTTAAAGCCGAAGC | 355 |
|  |  | R: ACATCAGAAGCACCTCCC |  |
| BIN1 | c.C593T | F: TGCTGCTAACCACGCTCAC | 664 |
|  |  | R: TGATGATGTCGCCTCCCTC |  |
| LPAR3 | c.G373A | F:TGCCTATGTATTCCTGATGT | 517 |
|  |  | R: CTTGCTTGGGTCCTCTTA |  |
| DDR1 | c.C2404T | F: GACCTTCTGTCGGTTCCCTT | 321 |
|  |  | R: AGTGGCGGCCAGTGTTCT |  |
| PARL | c.C153G | F:TGTGGTAGCCTACGCTTGT | 594 |
|  |  | R: CACTTGTCCCTGGGTCTG |  |
| SRC | c.C1337T | F: TGCCCTGTGCTCCCTACTC | 776 |
|  |  | R: GCCTAACATCAGACGCTCAACT |  |
| ROR2 | c.T1612C | F: TTGGCATCAGAGCCGTATTT | 1091 |
|  | c.G1687A | R: CGCCGAGCTGTTGTAGTTG |  |
| ARHGDIA | c.357_374del | F: AGGATGAGCACTCGGTCAACTAC | 800 |
|  |  | R: CAACTGCGGCACAAGGAAG |  |
| TNC | c.G434A | F: CACATCGCATCAACATCC | 594 |
| MMP10 | c.G1168A | R: ATCCCATCCTCTTTCCAG  F: AGTGAGTGCGAAGAAGGA  R: GTGGGTGTAGGGTAGGGA | 787 |
| MMP9 | c.G473T | F: CTATGGTTACACTCGGGTGGC | 965 |
|  |  | R: CGAAATAAGTGCGGGCTGA |  |
| C3 | c.T1474C | F: CAGGTCTCAGGGATTCGGGTGT | 391 |
|  |  | R: AGGGTGAGTGGCAGGGAACG |  |
|  | c.G3433A | F: TCAGACCGTTGTACCCAT | 844 |
|  |  | R: GTGTTCACCGTTTCCTGT |  |
| NLRP2 | c.C2342T | F: CTGAGGGTAGGAGTCGCTTGA | 807 |
|  |  | R: TGGCGGGTGCCTGTAATC |  |
| OSBPL5 | c.G1157A | F: GTGGAGACGCATTGGGAAGA | 776 |
|  |  | R:GGTCTGCGTGGTAGTAGTAGTCG |  |
| CENPB | c.1262_1264del | F: CTGCGACTACACCGCCAACT | 845 |
|  |  | R: GCACCTCATCACCATCCTCC |  |
| PER1 | c.C278T | F: GCCAACAGCAATGGTTCAA | 436 |
|  |  | R: GCCCACAGGGAAGAAAGAG |  |
| SLC13A1 | c.C814T | F: AAAGAGGTGGACTGCTGC | 470 |
|  |  | R: TTTCAAAGTGCTGGGATT |  |
| TYR | c.C346T | F: GGAGGTGGGAGTGGTATT | 923 |
|  |  | R: TGGGCTGAGTAAGTTAGGAT |  |
| REXO4 | c.192_206del | F: TGCGTGCTGAGGTATGCG | 1057 |
|  |  | R: AGGAATCTTCTTTCTCCCTGTCT |  |
| REXO4 | c.C976G | F: CCCTCCGTAGTGGCATTG | 493 |
|  |  | R: GGGTATCAGCACCTTCATTT |  |
| FSHR | c.C491A | F: TTGAGGCTACAGTGAGTTG | 1023 |
|  |  | R: CCCAGGCATACTTAGATAG |  |
| FKBP4 | c.C1066T | F: AGCAGTTGGCAAGAAGGA | 644 |
|  |  | R: CAGGTTAAGTGGAGGGATG |  |

Supplementary Table 3 Filtering process of whole-exome sequencing

| Filtering | No. of variants | No. of genes |
| --- | --- | --- |
| Nonsynonymous variant with frequency <0.1% in ExAC |  |  |
| All genes | 117620 | Exome |
| RPL-related genes | 284 | 95 |
| *In silico* predicted deleterious in RPL-related genes |  |  |
| Total | 35 | 28 |
| Missense | 26 | 22 |
| Nonsense | 3 | 3 |
| Frameshift | 1 | 1 |
| Non-frameshift | 5 | 5 |

ExAC: Exome Aggregation Consortium; MGI: Mouse Genome Informatics; RPL: recurrent pregnancy loss.
