# Supplementary material for: Exome-Sequencing Identifies Novel Genes Associated with Recurrent Pregnancy Loss in a Chinese Cohort
Source: Front Genet. 2021 Dec 2;12:746082. doi: 10.3389/fgene.2021.746082 (PMC8674582; doi:10.3389/fgene.2021.746082)

Patient 1
ADAMTS1:c.G1811A:p.R604H

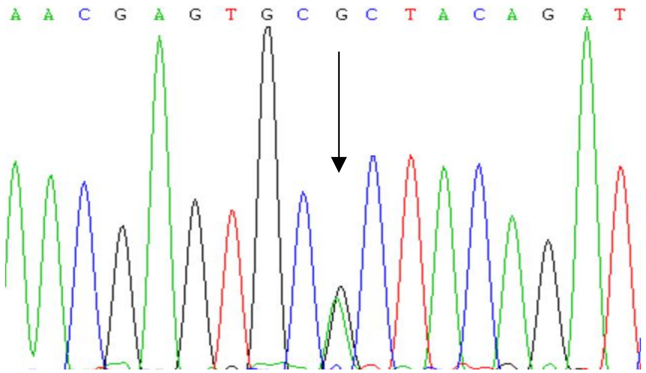

Patient 54
NOS3:c.G1507A:p.V503M

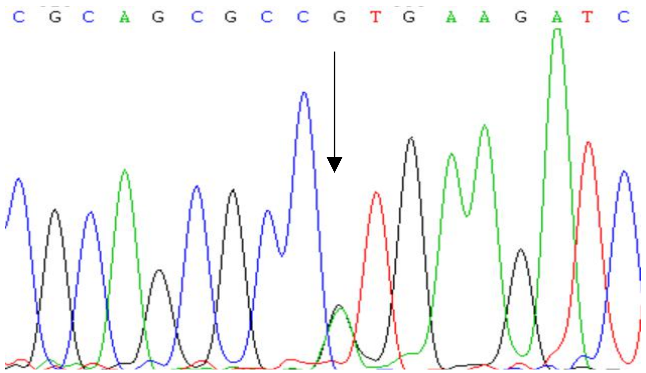

Patient 48
S1RP3:c.38delG:p.R13fs

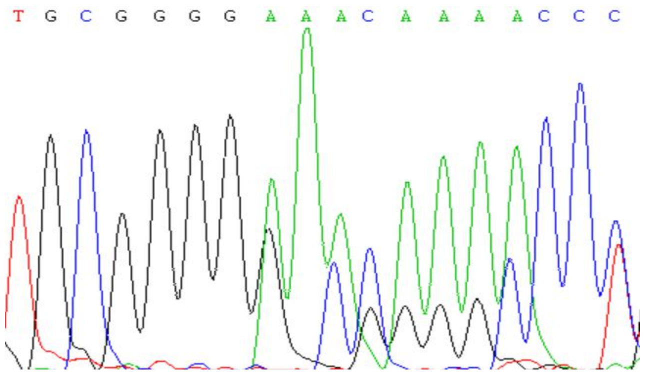

Patient 86
ASH1L:c.C7906T:p.P2636S

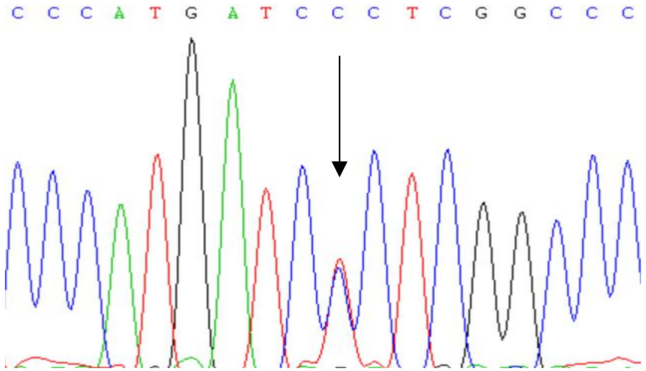

Patient 89
ASH1L:c.C1411T:p.R471W

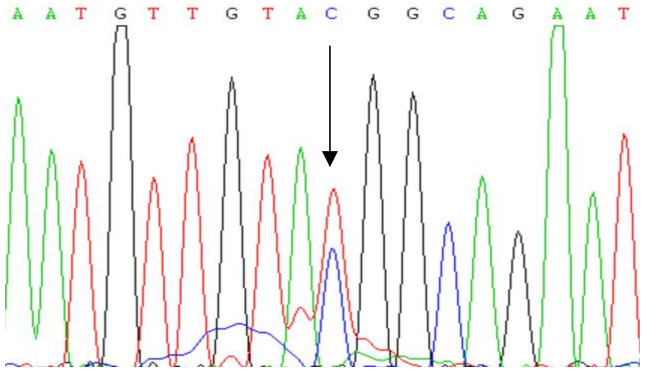

Patient 30
BIN1:c.C593T:p.T198I

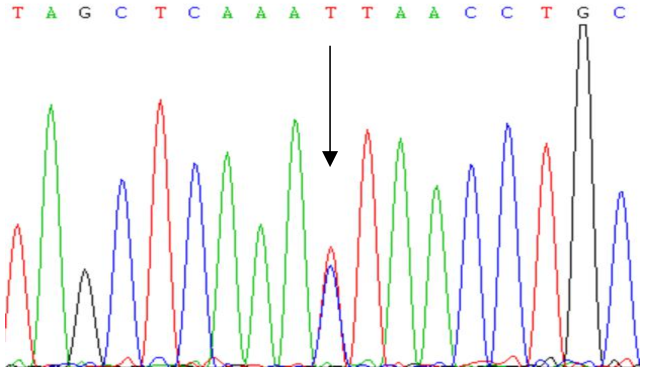

Patient 62
LPAR3:c.G373A:p.V125M

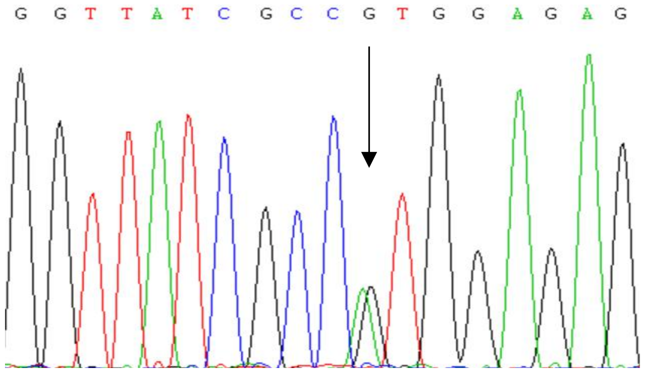

Patient 92
DDR1:c.C2404T:p.R802W

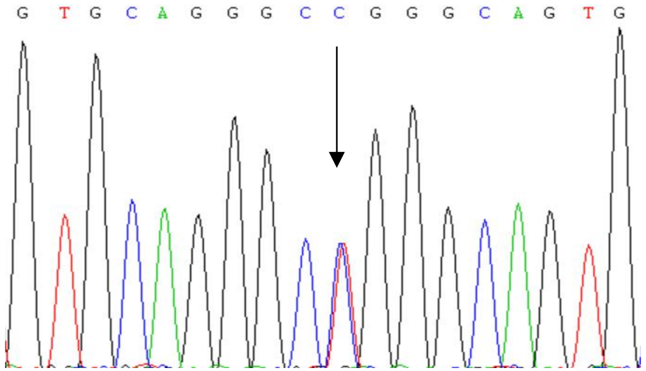

Patient 22
PARL:c.C153G:p.C51W

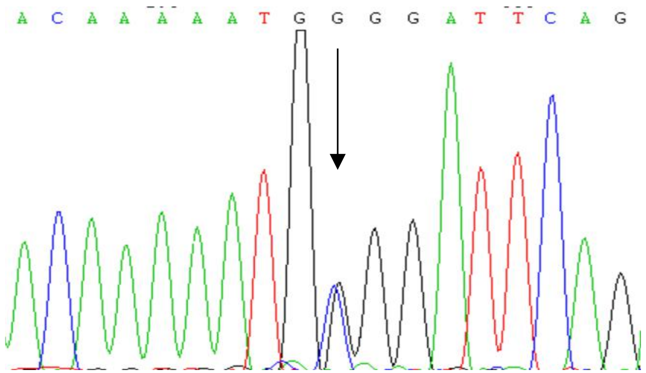

Patient 28
PARL:c.C153G:p.C51W

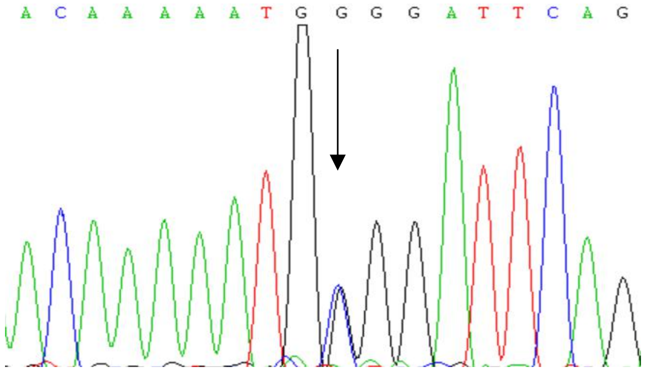

Patient 9
SRC:c.C1337T:p.S446L

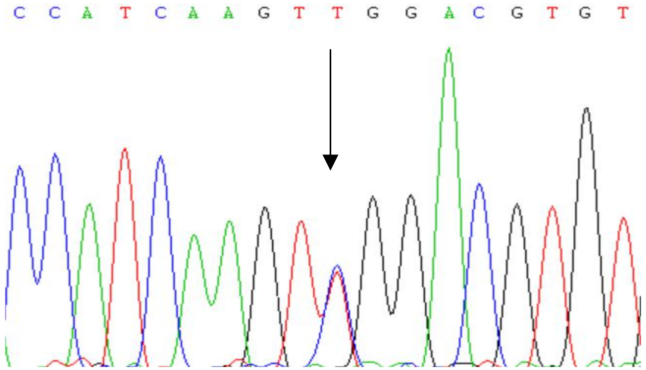

Patient 40
ROR2:c.T1612C:p.C538R

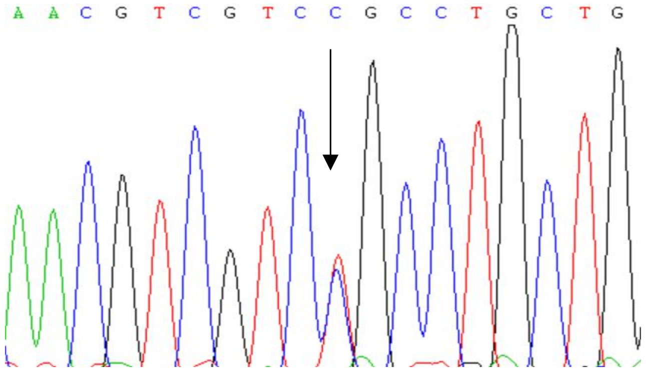

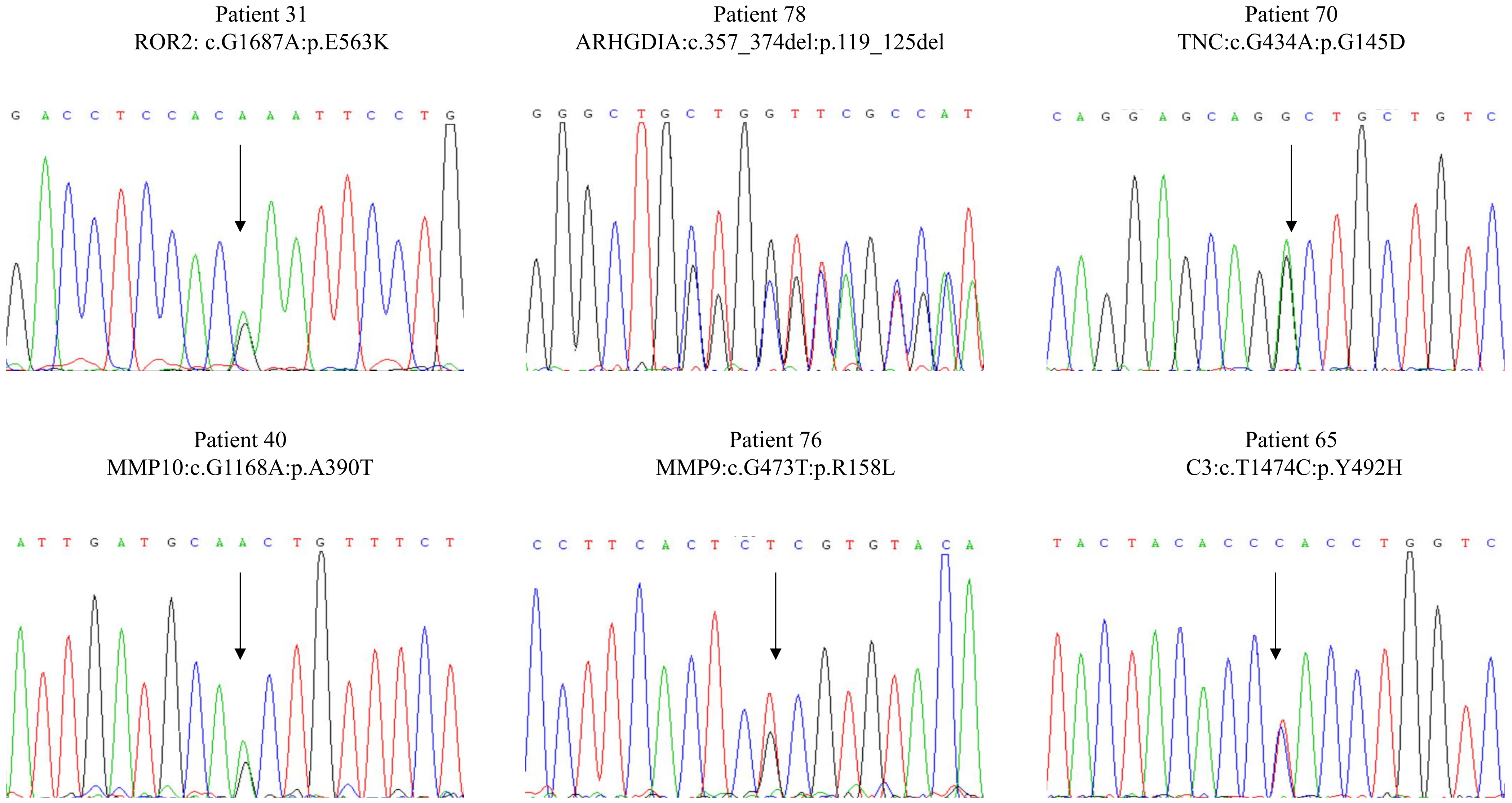
Patient 31
ROR2: c.G1687A:p.E563K
G A C C T C C A C A A A T T C C T G
Patient 78
ARHGDIA:c.357_374del:p.119_125del
G G G C T G C T G G T T C G C C A T
Patient 70
TNC:c.G434A:p.G145D
C A G G A G C A G G C T G C T G T C
Patient 40
MMP10:c.G1168A:p.A390T
A T T G A T G C A A C T G T T T C T
Patient 76
MMP9:c.G473T:p.R158L
C C T T C A C T C T C G T G T A C A
Patient 65
C3:c.T1474C:p.Y492H
T A C T A C A C C C A C C T G G T C

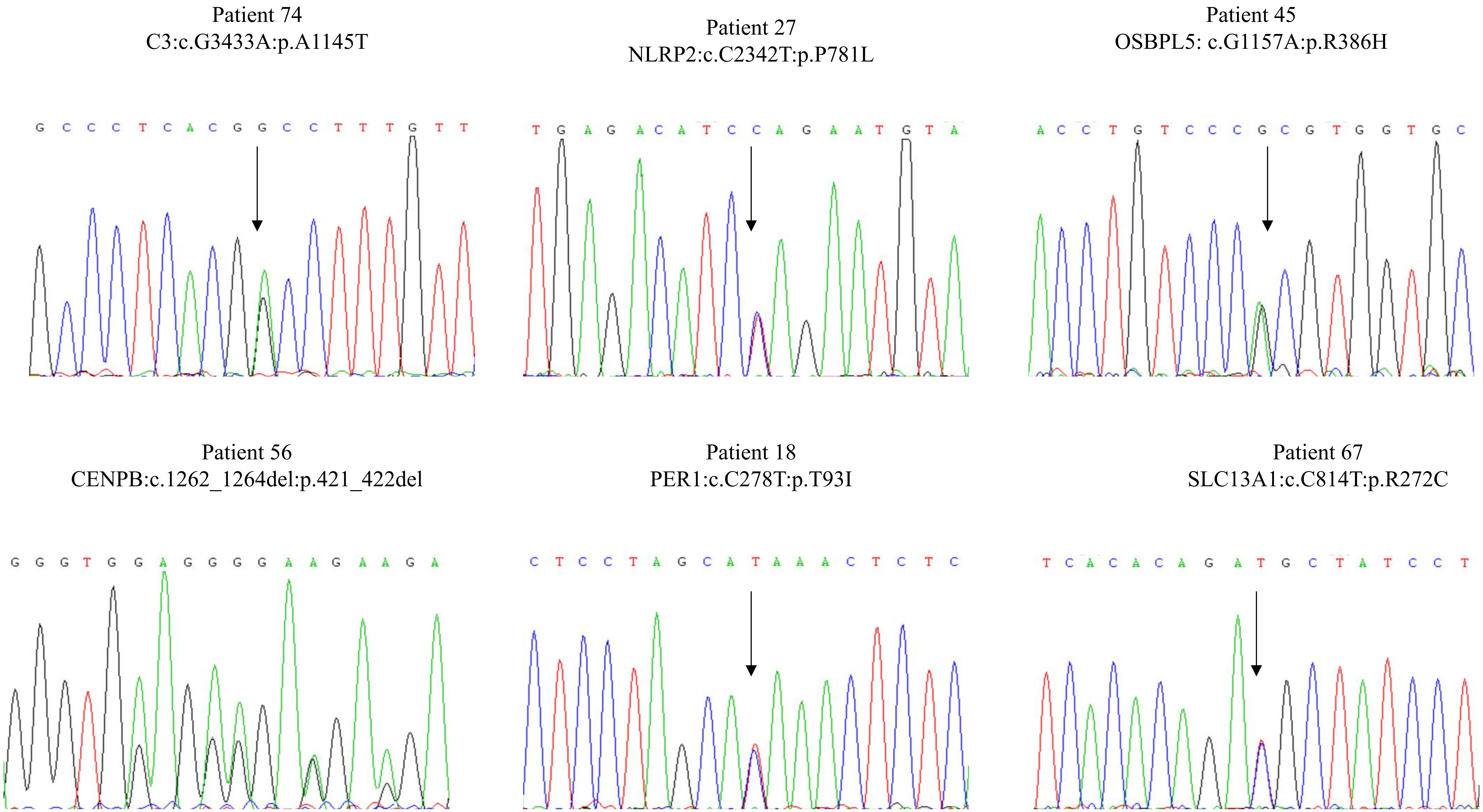
Patient 74
C3:c.G3433A:p.A1145T
G C C C T C A C G G C C T T T G T T
Patient 27
NLRP2:c.C2342T:p.P781L
T G A G A C A T C C A G A A T G T A
Patient 45
OSBPL5: c.G1157A:p.R386H
A C C T G T C C C G C G T G G T G C
Patient 56
CENPB:c.1262_1264del:p.421_422del
G G G T G G A G G G G A A G A A G A
Patient 18
PER1:c.C278T:p.T93I
C T C C T A G C A T A A A C T C T C
Patient 67
SLC13A1:c.C814T:p.R272C
T C A C A C A G A T G C T A T C C T

Patient 74
C3:c.G3433A:p.A1145T

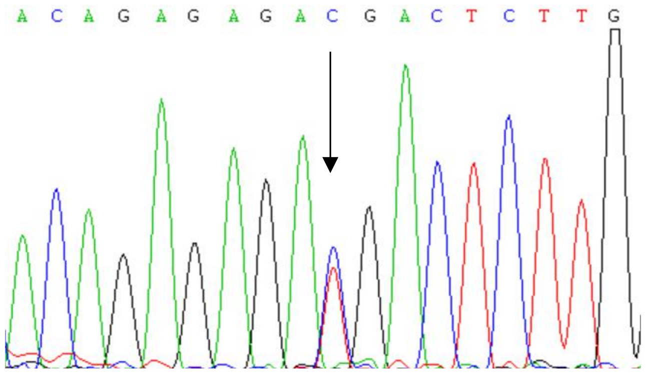

Patient 84
REXO4:c.192_206del:p.64_69del

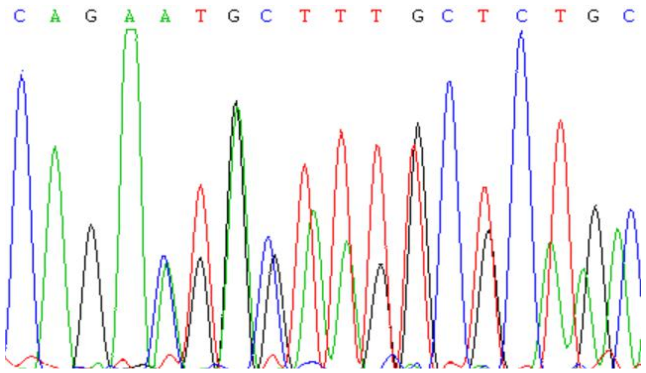

Patient 84
REXO4: c.C976G:p.H326D

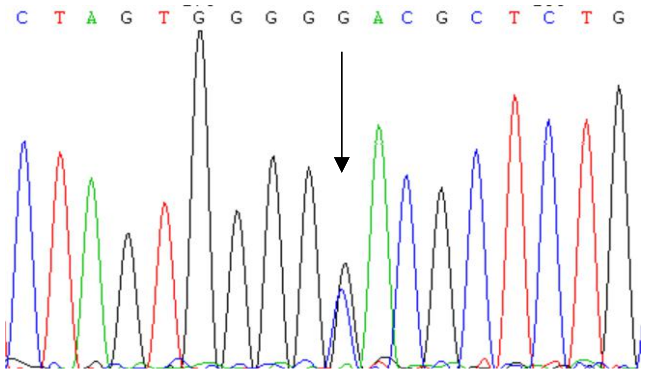

Patient 78
FSHR:c.C491A:p.S164Y

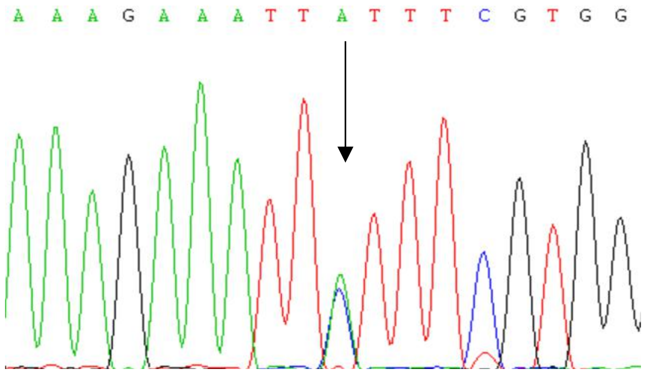

Patient 50
FKBP4:c.C1066T:p.L356F

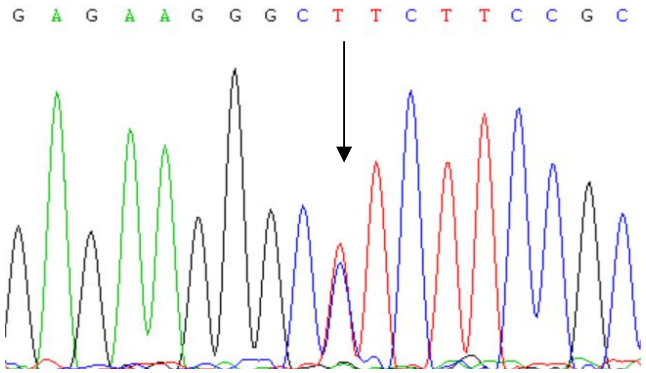

Supplement: Supplementary file 2 [file DataSheet1.PDF]
